# Australian experts' perspectives on a curriculum for psychologists working in primary health care: implication for Indonesia

**DOI:** 10.1080/21642850.2014.951937

**Published:** 2014-09-29

**Authors:** Diana Setiyawati, Grant Blashki, Ruth Wraith, Erminia Colucci, Harry Minas

**Affiliations:** ^a^ Global and Cultural Mental Health Unit, Centre for Mental Health, Melbourne School of Population and Global Health, The University of Melbourne, Melbourne, Victoria, Australia; ^b^ Faculty of Psychology, Universitas Gadjah Mada, Yogyakarta, DIY, Indonesia; ^c^ Nossal Institute for Global Health, The University of Melbourne, Melbourne, Victoria, Australia

**Keywords:** psychologists, primary mental health care, Indonesia, curriculum development, Australian experts

## Abstract

In Indonesia there is a pressing need to scale up mental health services due to a substantial unmet need for mental health care. Integrating psychologists into primary health care can potentially deliver affordable mental health services to communities and help to close the treatment gap. Australia is one of the pioneers in integrating mental health into primary health care, and the mental health reforms in Australia may have some implications for Indonesia. The aim of this paper is to examine the Australian experience and to reflect in particular on lessons that may be learnt to inform the development of curriculum for psychologists working in primary health care in Indonesia. Data were collected through semi-structured interviews with 12 Australian experts in primary mental health care. The focus of the interview was on the roles and skills of psychologists working in primary health care with a particular focus on the appropriate curriculum for psychologists. Overall, the Australian experts agreed that psychologists' roles and training should include both clinical skills and public mental health skills. The experts also agreed that psychologists should be able to educate the community about mental health issues and be capable of undertaking research and evaluation of programs. A central theme was the need for strong collaborations with general practitioners and existing agencies in the community so that psychologists are able to make appropriate referrals and also accept referrals. The lessons learnt from the Australian experience, which are most applicable to the Indonesian setting are: (1) the importance of adequate government funding of psychologists; (2) the value of evidence-based treatments such as Cognitive Behavioural Therapy; (3) the need to specifically train psychologists for primary care; (4) the need for flexibility in the psychologist workforce (e.g. location); and (5) the value of continuing supervision for psychologists to support them in their role.

## Introduction

Millions of people in Indonesia are affected by mental disorders. They do not affect only one specific group, but are prevalent in all communities and regions throughout the country. According to 2007 data in Indonesia, the prevalence of common mental disorders is reported to be 11.6% in the adult population (Balitbangkes, 2007).

Existing primary health care in Indonesia is currently a strong system based on widely dispersed local clinics. More than 9000 primary health care clinics serve more than 250 million Indonesian people, and they are located in every subdistrict of Indonesia. Every primary health care clinic includes approximately 30 staff who are responsible for providing care for approximately 30,000 people in each subdistrict (Abdullah, Hort, Abidin, & Amin, 2012; Daftar Puskesmas, 2011). Unfortunately, currently even the most basic mental health services are not yet established in most parts of Indonesia. In reality, the primary health care system does not generally provide mental health services *per se* in most cases (Irmansyah, Prasetyo, & Minas, 2009).

Historically, the mental health system in Indonesia has been centralised in mental hospitals. The first mental hospital was established in Bogor and Lawang by the Colonial Government in 1882. In 1966, the focus of mental health care was expanded to incorporate prevention, treatment and rehabilitation (Pols, 2006). Around a decade prior to the famous Alma Ata declaration on primary health care in 1978, the primary health care clinics in Indonesia (known as Puskesmas, the acronym of “Pusat Kesehatan Masyarakat” or the centre of community health) were set up as the backbone of the Indonesian health system (Hunter, 1996).

Currently, however, integration of mental health into primary health care is poorly developed (Setiawan et al., 2008). This slow progression can be understood due to a number of drivers, including the lack of health professionals and specifically leaders in charge of mental health programs. In addition, the workload of primary health care itselfs is already excessive (Hunter, 1996). Even though approximately 6% of all Indonesian civil servants are health workers, the ratio of mental health workers and the population is still far from ideal.

It is useful to compare the Indonesian and Australian mental health systems (see Table 1). Based on United Nations Development Programme (UNDP) world population prospect 2010 (Population Division of the Department of Economic and Social Affairs of the United Nations Secretariat, 2012), the predicted population per age group in Indonesia and the ratio of mental health workers are illustrated (Maramis, Van Tuan, & Minas, 2011), and are compared to Australia (Australian Institute of Health and Welfare, 2011). It can be seen that Australia with an overall population only about 10% of Indonesian society has a ratio of mental health workers to patients that is far higher than Indonesia, and this is also true for the psychologist workforces.

**Table 1.** The population demographic and availability of mental health services: a comparison of Indonesia and Australia.

Description	Indonesia	Australia
Total population	237,641,326^a^	23,058,110^b^
Percentage aged 0–24 (%)	45^c^	32.4^d^
Percentage aged 25–59 (%)	46.8^c^	49^d^
Percentage aged above 60 (%)	18.0^c^	25^d^
GNI per capita (US$)	2580^e^	43740^e^
Psychiatric beds per 10,000 population	0.4^f^	3.9^f^
Psychiatrists per 100,000 population	0.21^f^	14^f^
Psychiatric nurses per 100,000 population	0.9^f^	53^f^
Social workers per 100,000 population	1.5^f^	5^f^
Psychologists per 100,000 population	0.3^f^	5^f^

^a^Data from Badan Pusat Statistik (BPS) 2010/BPS-Statistics Indonesia (Badan Pusat Statistik (BPS-Statistics Indonesia), 2012).

^b^Data from Australian Bureau of Statistics (ABS) 2013 (Australian Bureau of Statistics, 2013).

^c^Data from Population Division of the Department of Economic and Social Affairs of the United Nations Secretariat 2010 (Population Division of the Department of Economic and Social Affairs of the United Nations Secretariat, 2012).

^d^Data from ABS 2010 (Australian Bureau of Statistics, 2012).

^e^Data from UNdata 2010 (GNI per capita (USD), 2010).

^f^Data from Mental Health Atlas 2005 (World Health Organisation, 2005).

This study has focussed on the Australian experience as a useful model for the Indonesian setting for several reasons. First, Australia is one of the pioneers in integration of mental health services in primary health care. This has been acknowledged by WHO and WONCA (World Health Organisation & World Organisation of Family Doctors [WONCA], 2008). Second, the process and culture of mental health reform could be a useful example for Indonesia. Third, Australia and Indonesia already have high-level collaborative links in terms of national mental health reforms and leadership. For example, the International Mental Health Leadership Program, taught in Australia and Indonesia, has 66 alumni (including the principal investigator), many of whom are in influential policy and academic positions and are leading mental health system reforms in Indonesia (Minas, 2012).

Turning now to the Australian setting, according to the Australian Institute of Health and Welfare, seven million Australians will experience a mental disorder over their lifetime (Australian Institute of Health and Welfare, 2011). This translates to 45% of the Australian population aged 16–85 years, and these conditions account for 13% of the total burden of disease (Ahmed, Gordon, & Ragg, 2011). Major reforms to improve access to high quality mental health care have been underway in Australia over the last few decades (Winefield & Chur-Hansen, 2004).

Integration of mental health into primary health care, as recommended by WHO, is a key strategy to close the treatment gap (World Health Organisation, 2001). Of note, Australia implemented this recommendation in the National Mental Health Plan for 2003–2008 (Australian Health Ministers, 2003). Thus, Australia is among 12 countries in the world that have been recognised as having best practice examples of integrating mental health into primary health care (World Health Organisation & World Organisation of Family Doctors [WONCA], 2008).

A feature of the policy reforms in Australia has been recognition that a collaborative team approach, consisting of diverse health workers, is needed to support the integration of mental health into primary health care. A well-funded program to support primary mental health care called the Better Outcomes in Mental Health Care (BOMHC) initiative was implemented by the Australian Government in 2001 (Pirkis et al., 2006; Winefield & Chur-Hansen, 2004). A key element, the Access to Allied Psychological Services (ATAPS), is one component of BOMHC, which supports psychologists and general practitioners (GPs) to collaborate to provide mental health care in particular evidence-based psychological treatments (Pirkis et al., 2006). Furthermore, additional funding under the Better Access program, which commenced in November 2006, has provided even more access to primary care psychological treatments. Under this funding scheme, patients referred by their GPs have Medicare subsidised access to psychologists and other allied health providers (Australian Institute of Health and Welfare, 2011).

From the psychologists' perspective, the integration of psychologists into primary health care through ATAPS funding has provided a rewarding professional experience (Winefield & Chur-Hansen, 2004). The project also showed significant positive clinical outcomes for 86% of people who participated in the program (Fletcher et al., 2009). Similarly, a concurrent Australian reform, the Better Access Program, also seems to have improved access to psychological treatments for people in the Australian community. According to the National Mental Health Survey in 2007, after the implementation of the Better Access Program, compared to 1997 surveys, patients with mental illness were more likely to be seeking specialist help in mental health care (Pirkis, Harris, Hall, & Ftanou, 2011).

Retouring then to the Indonesian context, clearly psychologists are crucial members of the mental health workforce, so there is potential capacity for greater mental health service provision. In fact, the Indonesian Psychological Association has approximately 9100 members and there are more than 82 Faculties of Psychology throughout Indonesia (Himpunan Psikologi Indonesia [HIMPSI], 2012). However, most of those Indonesian psychologists are working in private practice, so their services are not affordable or accessible for the broader population. Therefore, there is still a great need for better training and integration of psychologists into primary health care in Indonesia.

As an emerging reform in Indonesia, it is instructive to understand the genesis of psychologists working in primary health care in the Indonesian context. The initiative to integrate psychologists into primary health care was first taken by the Sleman District Government (a district within Yogyakarta Province) in collaboration with the Faculty of Psychology, Universitas Gadjah Mada in 2004. It was the first pilot project funded by the Local District Government. Starting with only six psychologists in 2004, the project has since expanded to include 25 psychologists, which translates to one psychologist for each primary health care clinic. The expanded pilot project program for the Yogyakarta Municipality (under the same Province) commenced in July 2010 (Retnowati, 2011).

The feedback from these programs indicated that psychologists faced some particular difficulties working in the primary health care setting. For instance, other primary health care providers had some confusion about the roles and skills of the psychologists. There was a sense that their training curriculum had prepared them to work in a specialty care clinic setting and not primary health care (Retnowati, 2011).

As an evolving field, there is no research evidence to date that can directly inform the development of a core curriculum for psychologists working in Indonesian primary health care. Thus, this research aims to investigate the types of roles and skills likely to be needed by psychologists working in Indonesian primary health care by exploring the Australian experience. The data will inform the development of the curriculum for psychologists working in primary health care in Indonesia.

## Methods

### Design

To gain an understanding of the Australian perspectives on psychologists working in primary health care, interviews with 12 Australian experts were conducted. The interview questions were focused on the roles of psychologists in primary care; skills needed by psychologists working in primary health care; and the appropriate education and clinical training for psychologists working in primary care. At the beginning of the interview, the interviewer explained the background and the aims of the study, including the current conditions of the Indonesian mental health system. Then, the interviewer asked the participants to respond to interview questions based on their experience in Australian systems. Prior to commencement of the study, the interview guide was developed based on the aim of the study, then piloted with four Australians from the mental health field. Revisions were made based on the feedback from the pilot process. More information about the specific questions asked of participants has been previously reported (Setiyawati, Blashki, Wraith, Colucci, & Minas, 2014).

This research received approval from the Human Research Ethics Committee, The University of Melbourne (Project No. HREC 1034956).

### Participants

The participants were Australian experts in primary mental health care across a range of disciplines, who were directly or indirectly involved in the integration of psychologists in primary health care. Participants were identified through one of the following methods:

1. *Publication*: The principal investigator reviewed the literature on primary mental health care and recorded the names of authors who were frequently published or cited by others. The principal investigator contacted these authors to seek voluntarily participation in the semi-structured interviews.
2. *Snowball sampling*: After those contacted agreed to participate, the principal investigator asked them for recommendations for other potential participants to be invited.
3. *Recommendation from a key professional organisation*: Recommendations were sought from the Australian Psychological Society (APS) as the lead professional organisation in this field in Australia.

Since there is no uniform agreement in the literature on an optimal sample size for qualitative research (Beitin, 2012), the number of participants in this research were recruited until it reached “data saturation”. Whether data saturation had been reached was based on stopping criterion (Francis et al., 2010), notably that no new themes were emerging from the interviews.

The background of participants in the study were psychologists working in primary health care, psychologists working in rural/remote/indigenous area, a representative from the APS, a psychiatrist, a senior lecturer of psychology, a researcher in mental health policy, a GP and a cross-cultural expert who has in-depth understanding of Australian and Indonesian cultures.

All identified participants were contacted by sending a formal letter of invitation and a consent form. The interviews were then conducted at a convenient time and place for each participant. Twelve experts agreed to be interviewed by the principal investigator and to be tape-recorded during the interview. Data were being analysed concurrently and after the 12 experts being interviewed and there were no new themes emerging, recruitment of participants was stopped. Audit trail by writing field notes on how the research was conducted and what the principal investigator hears, sees and thinks during the fieldwork was conducted to ensure the quality.

### Data analysis

The data collected were transcribed verbatim and analysed using a thematic approach. The Nvivo 10 software (QSR International, 2012) was employed to manage the data and to facilitate the coding process. The analyses were conducted based on a process proposed by Liamputtong (2009). It started with open coding, with the principal investigator examining any new relationships between the data. The next step was axial coding or a conceptual coding process. This process involved reassembling the data in order to identify the connection between category and subcategory. The final step was selective coding. This was aimed to establish links between the codes and to find a meaningful connection between coding. The principal investigator presented the data and the coding to the research team before deciding the final thematic categorisations.

## Results

The results are presented below and categorised based on important roles and skills of psychologists in relation to (1) patients, (2) other primary health care providers, (3) primary health care as service providers, (4) community and (5) policy.

### Roles and skills in relation to patients

Most participants mentioned that the basic role, and skill, of psychologists in primary health care is providing clinical psychological services. These ranged from conducting assessments to providing treatments. In general, there are four main findings on roles and skills in relation to patients: (1) capability to handle general community populations; (2) assessment skills; (3) diagnostic skills; and (4) knowledge and skills in providing treatment.

Capability to handle general broad populations was a strong theme. For example one participant stated that “psychologists largely practice very much like generalists” (P^1^-8^2^). This means that psychologists should be capable of dealing with patients from all ages, ranging from “children problems and adolescence” (P-5), to “adult” (P-3). “They might have to work with young children as well as very older people, so they have got to be able to communicate very well” (P-12). They also have to face various problems that range from “common mental health disorders” (P-6) to “common chronic illness” (P-2), and “not just one kind of a problem” (P-12).

Assessment skill was another important role. For example one participant stated that “the assessment is really the psychologists’ role” (P-10). The participants also highlighted that in conducting an assessment, psychologists should “be able to conduct a comprehensive assessment,” (P-1) and should also understand “how to assess people with various kind [or aspects] of assessment, like mental health status, or cognitive status, so they are trained to do an assessment” (P-9). Furthermore, the participants pointed out that conducting assessments should not only employ existing assessment tools, but should also include developing tools tailored to a patient's culture. As one expert explained: “I'm using some Aboriginal measures I developed. In my research I'm developing Aboriginal measures” (P-3).

Providing diagnosis was highlighted by the participants as the next important role or skill. For example, participants emphasised that psychologists should know “how to diagnose them” (P-9). Another participant pointed out the importance to “know what is going on diagnostically” (P-2). This skill is a basic foundation for the next skill, providing treatment.

Knowledge and skill in providing treatment that matches the diagnosis was considered as very important, as demonstrated in these quotes: “to match whatever the disorder was with the best treatments for these disorders” (P-9) and “knowing what are specific treatments are for this specific diagnosis” (P-4). Specifically, psychologists should be able to “deliver these evidence-based treatments” (P-12), as well as “provide effective and appropriate treatments” (P-1). In primary health care, the treatments should contain “some really concrete strategies” (P-3). Another participant explained in detail:

The other set of skills then is treatment, so there are concrete interventions, strategies, that could be offered immediately to take the pressure off, and then subsequently to that, we might help them change their thinking pattern that might have led to this. So, I wouldn't see this as either/or. I would start with a very concrete strategy, to help the person gain some control over their anxiety, or their fear, or their mood, and then we would go back to the other stuff later. (P-12)

### Roles and skills in relation to other primary health care providers

There are two main themes about psychologists' roles and skills in relation to other primary health care providers: (1) inter-professional communication and (2) recognising the unique contribution of psychologists. Most participants agreed that communication is the key to building a good relationship between psychologists and other primary health care providers. One expert mentioned the importance of the “ability to communicate effectively with other professionals” (P-1) and another expert highlighted that “we need a standard relationship skill” (P-8). Communication with other health professionals includes an understanding of how to refer and accept referrals. One of the participants highlighted that psychologists should:

 … know how to connect with those people and develop relationships, also the appropriate way to refer to other people. You need to know how to speak the right language and know the right words to get another service to accept your patient, to accept the referral. I think those sort of skills are really important. (P-6)

To communicate with other primary health care providers, especially GPs, psychologists should be supportive and employ different modes of communication including letter and telephone. This skill will help to “maintain the flow of referrals with the GP” (P-1). One participant mentioned that psychologists have to be capable of:

 … providing the consumers with the service, and communicating back to the GP. You know what has happened and what else you'd recommend for the client. But I think sometimes it's up to the GP and the psychologists. So I guess if you know the GP well, they might be supportive, communicate through the telephone, make telephone calls, write letters, they are concerned about risk to the client. (P-1)

The ability to emphasise the unique strength of psychologists and to recognise that they are equal to other Psychologists should be self-confident in promoting their unique skill set professionals is also very important. One participant highlighted this:

I think the capacity to highlight the unique strength of psychologists, is critical. And also the capacity to feel equal and to communicate equally with doctors. Some people still feel intimidated by doctors, and I think that's ridiculous. We've got to develop a sense that they are our colleagues, friends, and that we're all equal. We are doing things differently. (P-10)

### Roles and skills in relation to primary health care as service providers

There are two main themes that emerged regarding the roles and skills of psychologists in relation to primary health care service providers: (1) analysis and evaluation skills; and (2) managing stigma.

Analysis and evaluation skills are clearly very important to contribute to primary health care as service providers. One participant highlighted that psychologists should be capable to “analyze suicide, social-emotional wellbeing, health and mental wellbeing” (P-3). Two participants expressed that psychologists also should be very capable of “conducting evaluation” (P-6) and “program evaluation” (P-9). The analysis skill would give psychologists strong basics to develop a mental health program and the evaluation skill would help psychologists to develop effective primary health care service.

Managing the stigma of mental illness is also another important theme, as stigma can be a big barrier in accessing psychological services. One participant stated that “barriers can be from patients, which can be the stigma”. Seeing a psychologists can be perceived as “if you see a psychologist it means you're mad”. Another participant stated, “ … fear that they are going to get inside and change my mind. And there is, I think, quite a lot of fear about what psychologists do” (P-2). Finally one interviewee expressed, “I would equally feel that if psychology can remain in primary care rather than having to be sent off to some sort of super specialist practice, it would overcome some of that stigma” (P-2). Psychologists working together in primary health care can reduce that stigma of mental illness.

### Roles and skills of psychologists in relation to the community

Three themes arose regarding psychologist's role in the community; (1) contributing to public health; (2) respecting different cultures and (3) promoting familiarity with existing services in the community. One participant stated, “contribute to public health is important” (P-10) and another noted that one role for psychologists is “ … providing a bit of health education” (P-5). These statements suggest that study participants recognised that psychologists have a responsibility not just to individual clients, but also to the larger community. As a consequence, psychologists have to have a public health perspective.

Respecting culture was also viewed as an important skill for psychologists. Psychologists should understand that mental health problems are perceived differently in different cultures and this also has implications for service design, for example:

In Aboriginal culture, the way we present to community relations, we call it a family counselling service. We don't call it mental health. As the family counselling service, we present it in more holistic manner so we have adolescents and children upstairs, adult downstairs. And we try to provide integrated approach, where that means we have GPs, family counselling service, and psychiatrists. (P-3)

Familiarity with existing services in the community is also very important, so that psychologists can appropriately refer clients to them. One participant highlighted this point: “I think a lot of this integrated primary work should understand about moving into the community and taking into consideration familiar alliances into the community by taking care of what already exists” (P-3). Respondents indicated that issues with accessing other basic necessities such as food, accommodation and finances should be addressed at the same time as mental health problems. Psychologists should understand that some patients might have complex needs that require social services to be in place. Therefore, the relationship to existing community services is very important:

If you're working out there and you don't know who's around you, or you don't know how to refer to these agencies or how to get your patients some food when you don't have anything in the cupboard, it's really very stressful. You can't do therapy and you can't treat somebody's depression if you have no food to eat for the weekend, you constantly need to link other people to the services. (P-6)

### Roles and skills in relation to policy

The main findings in relation to policy are that psychologists ought to have (1) good understanding health system and (2) a capacity to advocate for patients within the health system. Psychologists should have a good understanding of the mental health system and the general health system, such as payment scheme for psychologists: “Good understanding of all the different sources of funding, and how it works. So, understand(ing) the government policy” (P-6). Another participant highlighted that psychologists should have an “understanding not just [of] the mental health care system, but (of) the general health care system” (P-7). Therefore, psychologists could involve themselves in “advocacy and navigating health system to prioritise mental health” (P-4).

### Lessons learnt

Experts were asked to comment about the integration of psychologists into primary health care in Australia. There are five main findings that are most applicable to the Indonesian setting: (1) the importance of adequate government funding of psychologists; (2) the value of evidence-based treatments such as Cognitive Behavioural Therapy (CBT); (3) the need to better train psychologists for primary care; (4) the need for flexibility in the psychologist workforce (e.g. location); and (5) the value of continuing supervision for psychologists to support them in their role.

Most participants perceived that the implementation of government funding has facilitated the integration of psychologists in primary health care. Specifically, the newly introduced funding model helps patients to be able to afford and access psychologists. Experts highlighted that patients can now avail of sessions with a psychologist for a set number of times a year under the ATAPS funding:

ATAPS is 12 sessions per year, but, in exceptional circumstances, the GP can give a referral for another six, so they can get 18 sessions in a year, where in Medicare that just changed with the last federal budget, I think they can get 10 sessions. (P-6)

The funding especially encouraged evidence-based treatments and there is a strong focus on CBT as a psychotherapy approach. This appears to be the expectation of many of the GPs referring patients: “Some GPs will give you some more extent of information and others will just like say ‘anxiety-depression’, ‘provide them with CBT’ (P-1)”. Another expert also pointed out that CBT works very well in a limited number of sessions:

 … under Better Access and Better Outcomes, people use a lot of common behavioral therapy, CBT, and I think that works really well especially when you've got a time limited number of sessions, so you can equip people into six to twelve sessions, with specific skills. I think people do a lot of that. (P-6)

Even with these large scale reforms, insufficient training for preparing psychologists to work in primary health care is still an issue in Australia. One participant mentioned the benefits of primary care placement, as one aspect of the current training that needed to be improved:

Well, I think they need to have placement experiences in primary care, not only to learn, but to see, “Is this what I like?”, “Do I like to work in this place?”. Yes, I'd like to see more primary care placements. Because then we get opinions about what the work is like not from your lectures, but from your placement experiences. (P-9)

The integration of psychologists into primary health care in Australia does not necessarily mean that psychologists work together with GPs or other primary health care providers in the same clinics. However, one participant did highlight the benefit of co-location with GPs and other primary health care providers:

Well, the reality is that the Better Access model was designed to support GP and psychologists working together in primary health care, but what exactly happened is a lot of psychologists continue to work on their own solo; they are in a private practice setting, and while they are forced to communicate with the GP you know after the six sessions you are meant to write a report and so on. Basically good primary health care psychologists collocated with the primary care general practitioners, so that's my understanding of the model. (P-10)

The Australian system strongly encourages continuing supervision by senior psychologists: “It needs to be supervised, because when you're working in primary care, quite often you're working independently. So, I think supervision is very important and is encouraged in Australia” (P-1). The participant also highlighted that even psychologists have to find and pay their supervisors, and it is a very important aspect:

They have to find supervisor and they have to pay for supervision. We want more and more psychologists to have more and more continuing supervision, even when they are qualified, because you need supervisor, especially in the first months after you finish study. (P-9)

## Discussion

The participants in this study identified clear roles and skills for psychologists to successfully work in primary health care. These roles and skills highlighted by participants relate to their relationship to their patients, other primary health care providers, primary health service providers, policy and also the broader community. The broad spectrum of the roles and skills is consistent with the conceptual model proposed by Talen, Fraser, and Cauley (2005) based on their experience for 10 years in the Wright State University, where they overviewed similar domains of expertise for psychologists working in primary care settings. They concluded that psychologists should play as the roles of therapist, consultant, trainer and advocate. Thus, they should be able to deliver intervention, consultation, programming, research and advocacy.

This study also found a set of roles and skills for psychologists that are consistent with those previously proposed by McDaniel, Belar, Schroeder, Hargrove, and Freeman (2002). For example, understanding the local health policy and health care systems was considered essential.

The participants also highlighted the importance of inter-professional collaboration in primary health care. This collaboration includes the ability to communicate through multiple channels such as letter and telephone. The participants also emphasised that psychologists should feel equal to other health professionals and should be feel confident that they can make their own contribution to clinical care. These points are consistent with previous research of integrated care with interpersonal and communication skills highlighted in research by Bray (1996) and Bluestein and Cubic (2009). Notably, understanding cultural diversity, which was identified by Bluestein and Cubic (2009), was also a key theme found in this study. In the Australian context, dealing with multicultural backgrounds and Indigenous people is a particularly crucial skill.

The main lessons that can be drawn from Australian experiences to be implemented in Indonesian were: (1) the importance of adequate government funding of psychologists; (2) the value of evidence-based treatments such as CBT; (3) the need to better train psychologists for primary health care; (4) the need for flexibility for the psychologist workforce (e.g. location); and (5) the value of continuing supervision for psychologists to support them in their role.

The importance of funding was recognised as an essential factor to encourage the integration of psychologists into primary health care. There was a strong appreciation of the importance of the ATAPS and BOMHC funding in Australia as a critical step. In the US context, researchers have identified that funding is recognised as a key starting point for mental health providers' willingness to accept patients (Trude & Stoddard, 2003). In reality, funding will never be enough for any type of health system; therefore the management of the health budget always involves trade-offs. Often it is the case that allowing flexibility at a local level allows those with local knowledge to get the best outcomes for the money available.

The mental health budget in Indonesia was less than 1% of the total health budget and 97% of it was allocated to mental hospitals (Setiawan et al., 2008). The budget for mental health programs in primary health care is therefore limited. Fortunately, the Indonesian Government system is characterised by centralisation, which co-exists with decentralisation in which provinces or districts have a strong control over health expenditure (Ramesh & Xun, 2008). This condition could maximise the flexibility for each province or district to create innovations for their local health system.

The policy to accommodate the needs in improving access to psychological therapies was carefully drawn from a research that involved consumers. It resulted in recommendations such as value of evidence-based treatments, especially CBT (Pilgrim & Carey, 2012). Consumer advocacy is very strong in Australia (Moulding et al., 2009). In fact, the current Australian Government policy states that consumers and carers must be involved in the mental health reforms. Their active involvement includes planning, implementation and evaluation of mental health services reforms (Alliston, Kluge, & Fudge, 2009).

In Indonesia, the human rights of mental illness patients are still overlooked. Some example of basic consumer rights that do not exist in Indonesian law include protection of the right of the consumer, the principle of least restrictive treatment, and informed consent and confidentiality (Irmansyah et al., 2009). Involving consumers to listen to their life experiences is still in its infancy in Indonesia and should be a priority for the next movement.

The awareness to build stronger psychologist workforces has been raising discussions in universities (Retnowati, 2011). The need to better train psychologists for primary health care, the need for flexibility for the psychologist workforce (e.g. location) and the value of continuing supervision for psychologists are some points highlighted from this study, which will be a valuable guidance for future direction of workforce development.

The limitation of this study is the possibility of sample bias as the recruitment process was based on publication and snowball sampling; therefore academicians dominated the composition of participants. In addition, obviously there are differences between Australia and Indonesia in terms of legal, policy and culture. It might influence the analysis. Involving cross-cultural experts as participants and understanding the context and background of both countries are among the efforts to minimise this limitation.

## Conclusion

Based on Australian experience, this study highlighted the fact that the roles and skills of psychologists in primary health care include many basic aspects of clinical care. Psychologists also need to have the skills to collaborate with other primary health care providers and provide a unique strength to the primary health care team. In addition, psychologists should contribute to public health in the community and be familiar with services for patients in the community. Finally, psychologists need to have an understanding of the general health system in order to advocate for funding for mental health services.

Since there is no research evidence that specifically relates to the roles and skills of psychologists working in primary health care in Indonesia, the Australian experts' perspectives will be used as a starting point to inform the development of curriculum that is applicable to the Indonesian context. Valuable lessons learnt from the Australian experience in integration of psychologists into primary health care are: (1) the importance of adequate government funding of psychologists; (2) the value of evidence-based treatments such as CBT; (3) the need to better train psychologists for primary care; (4) the need for flexibility for the psychologist workforce (e.g. location); and (5) the value of continuing supervision for psychologists to support them in their role. The application to the Indonesian context needs careful attention due to different legal, policy and culture. Therefore, two further studies were conducted to follow up this study (Setiyawati, Blashki, Wraith, Colucci, & Minas, 2013; Setiyawati et al., 2014).

## Notes

1. P, participant code.

2. Participant's number in data coding.
